# Implementing Serosurveys in India: Experiences, Lessons Learned, and Recommendations

**DOI:** 10.4269/ajtmh.21-0401

**Published:** 2021-10-04

**Authors:** Alvira Z. Hasan, Muthusamy Santhosh Kumar, Christine Prosperi, Jeromie Wesley Vivian Thangaraj, R. Sabarinathan, V. Saravanakumar, Augustine Duraiswamy, Ojas Kaduskar, Vaishali Bhatt, Gururaj Rao Deshpande, Padinjaremattathil Thankappan Ullas, Gajanan N. Sapkal, Lucky Sangal, Sanjay M. Mehendale, Nivedita Gupta, William J. Moss, Kyla Hayford, Manoj V. Murhekar, for the IMRVI Study Group

**Affiliations:** ^1^ International Vaccine Access Center, Department of International Health, Johns Hopkins Bloomberg School of Public Health, Baltimore, Maryland;; ^2^ ICMR-National Institute of Epidemiology, Chennai, India;; ^3^ Diagnostic Virology Group, Indian Council of Medical Research (ICMR)–National Institute of Virology, Pune, Maharashtra, India;; ^4^ Encephalitis Group, ICMR–National Institute of Virology, Pune, Maharashtra, India;; ^5^ World Health Organization, Southeast Asia Region Office, New Delhi, India;; ^6^ Division of Epidemiology and Communicable Diseases, Indian Council of Medical Research, New Delhi, India;; ^7^ Department of Epidemiology, Bloomberg School of Public Health, Johns Hopkins University, Baltimore, Maryland;; ^8^ W. Harry Feinstone Department of Molecular Microbiology and Immunology, Bloomberg School of Public Health, Johns Hopkins University, Baltimore, Maryland

## Abstract

Serological surveillance for vaccine-preventable diseases, such as measles and rubella, can provide direct measures of population immunity across age groups, identify gaps in immunity, and document changes in immunity over time. Rigorously conducted, representative household serosurveys provide high-quality estimates with minimal bias. However, they can be logistically challenging, expensive, and have higher refusal rates than vaccine coverage surveys. This article shares lessons learned through implementing nine measles and rubella household serosurveys in five districts in India—the challenges faced, the potential impact on results, and recommendations to facilitate the conduct of serosurveys. Specific lessons learned arose from challenges related to community mobilization owing to lack of cooperation in certain settings and populations, limitations of outdated census information, nonresponse due to refusal or unavailability during survey enumeration and enrollment, data collection issues, and specimen collection and handling issues. Although some experiences are specific to serosurveys in India, these lessons are generalizable to other household surveys, particularly vaccination coverage and serosurveys conducted in low- and middle-income settings.

## INTRODUCTION

Serological surveillance for vaccine-preventable diseases, such as measles and rubella (MR), can provide direct measures of population immunity across age groups, identify gaps in immunity, and document changes in immunity over time.^1^^,^^2^ Evidence from serological surveillance can be used to guide vaccination programs, including evidence to tailor the age range of vaccination campaigns and identify geographic areas for targeted vaccination activities.^1^ However, household surveys with blood collection can be logistically challenging, expensive, and potentially have high refusal rates.^2^ Despite the challenges, rigorously conducted, representative household serosurveys generate high-quality seroprevalence data, one of the best estimates of population immunity. Seroprevalence data are increasingly used to monitor progress toward goals such as polio eradication and measles, rubella, and hepatitis B elimination, as well as recent efforts to assess the prevalence and transmission dynamics of SARS-CoV-2.^1^^,^^3^^–^^5^

High-quality household serosurveys are anchored in the inclusion of a representative sample of the target population, usually by enrolling a probability-based sample. A probability-based sample requires that every eligible respondent has a known and nonzero probability of selection. The WHO Vaccination Coverage Cluster Surveys Reference Manual, a commonly used survey tool adapted from the Demographic and Health Survey manuals, was updated in 2018 and substantially modified the methods to enable surveys to generate quantifiable probabilities of selection and weight them appropriately to estimate vaccination coverage in the target population.^6^^,^^7^ These changes included steps to map and enumerate all people in the cluster, random selection of households by a central team instead of interviewers in the field, elimination of enrollment quotas and residency requirement, and documentation and use of survey weights in the analysis. However, implementation of these recommendations remains challenging, and they do not fully address nonsampling errors, which can also affect the precision and representativeness of the survey results.

Prior publications have described implementation challenges of household vaccination coverage surveys, including sources of nonsampling error, methods to reduce them, or issues to consider when interpreting surveys because nonsampling errors cannot always be avoided.^8^^–^^12^ Several studies also described best practices when nesting a serosurvey within an established coverage survey or implementing a combined Multiple Indicator Cluster Surveys/National Immunization Coverage Survey.^2^^,^^13^^,^^14^ Furthermore, recently developed WHO measles and rubella serosurvey guidelines provide guidance on how serosurveys can be used to support countries to achieve their measles and rubella elimination goals.^3^ However, published information related to lessons learned from implementing stand-alone household serological surveys are scarce.

Nine serosurveys were conducted in five districts in India to estimate district-level seroprevalence against measles and rubella before and after an MR vaccination campaign. We documented challenges encountered during serosurvey implementation to better understand how they contributed to nonsampling errors in the results. Here we describe the challenges and their potential impact on study findings and provide recommendations to minimize these challenges and errors when implementing household serosurveys. Although some experiences are specific to serosurveys in India, these lessons are generalizable to other household surveys, particularly vaccination coverage and serosurveys conducted in low- and middle-income settings for a broad range of pathogens.

## MATERIALS AND METHODS

**Figure 1.**
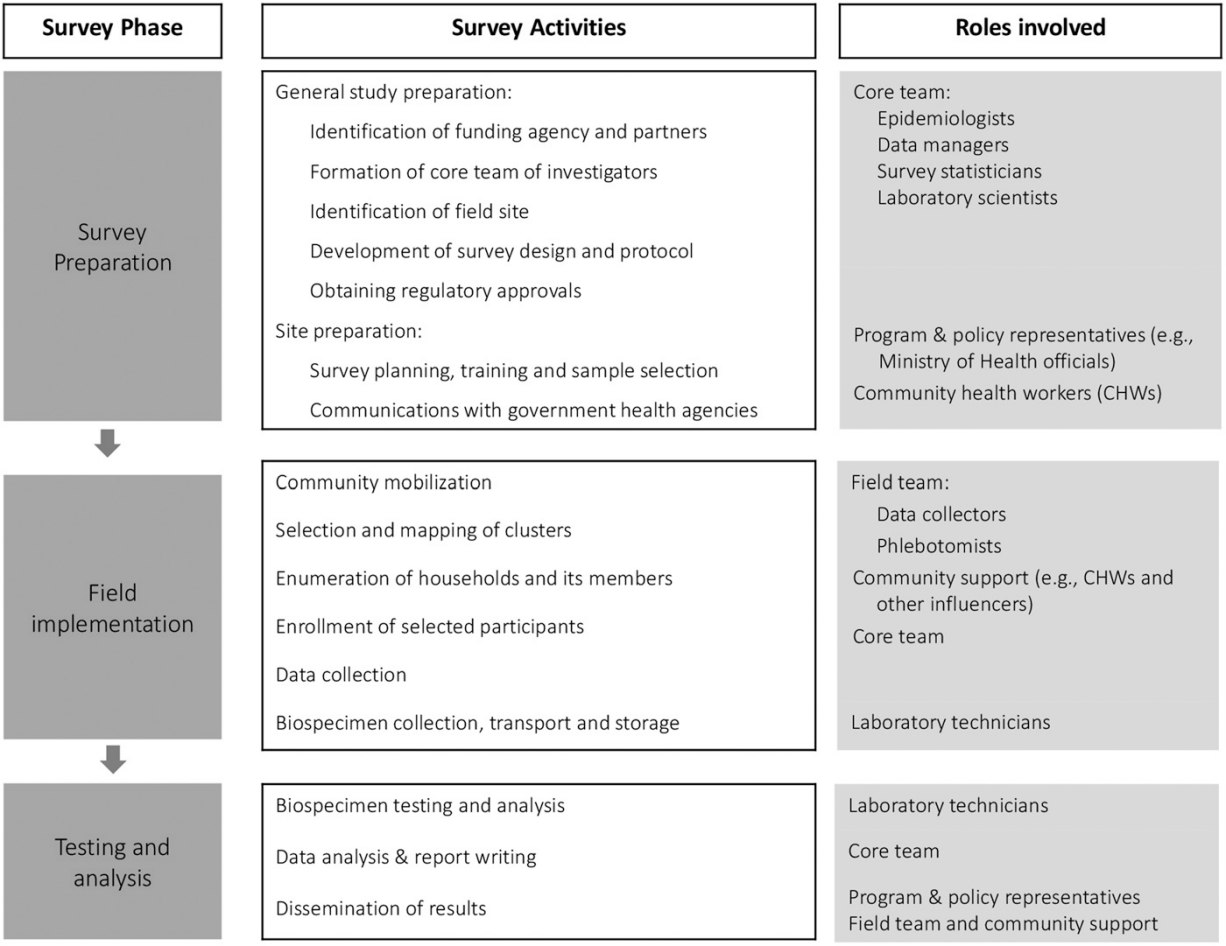
Overview of household serosurvey phases, activities, and roles involved.

The serosurveys were conducted from 2018 to 2020 in five districts of India among three age groups: children 9 months to < 5 years, 5 to < 15 years, and women 15 to < 50 years (Supplemental Appendix). Each survey followed the same three phases: survey preparation, field implementation, and testing and analysis (Figure 1).

### Survey preparation phase.

This began with study preparation, including identifying a funding source; formation of a core team of investigators comprised epidemiologists, data managers, survey statisticians, and laboratory scientists; identification of field sites; and development of protocols in collaboration with field site investigators. Site preparation, in which the core team and site investigators conducted survey planning, field team training, and selection of the probability sample followed. Communications with district and state program managers, policymakers, and community health workers (CHWs) were initiated to obtain permission and support.

A multistage sampling design was implemented using guidance from the WHO Vaccination Coverage Cluster Surveys Reference Manual, for which the core team selected 30 villages or wards in each district for each serosurvey using the 2011 census followed by selection of one census enumeration block (CEB) or one cluster in each village or ward. The generic term “cluster” is used for a CEB, which is a well-defined area in a village or ward with 120 to 150 households as per the India census.

### Field implementation phase.

Site investigators and field teams led the field implementation phase with support from CHWs, local leaders, the core team, and other community members. Community mobilization was conducted throughout all steps of the survey. These steps involved mapping selected clusters and enumerating households and household members in the mapped area to determine the final sampling frame. Participants were randomly selected from the sampling frame based on study eligibility criteria (Supplemental Appendix). This was followed by enrollment of selected participants, data and blood collection, and transport and storage of the specimens.

### Testing and analysis phase.

The testing and analysis phase involved blood specimen testing, data analysis, report writing, and dissemination of results. Laboratory coordinators and technicians led testing. The core team of investigators completed data analysis and report writing. Finally, field teams disseminated results with support from policymakers and CHWs.

Challenges and lessons learned were documented at all steps of the field implementation phase of the surveys through near real-time data monitoring, oversight by site investigators, daily reporting from field teams through WhatsApp messaging service, weekly conference calls, and frequent monitoring site visits.

## RESULTS

In each serosurvey, 780 children (in both age groups) and 390 women (in the post-MR campaign serosurveys only) were selected to participate. Of those selected, 78% to 91% of children and 81% to 91% of women were enrolled across the nine serosurveys. Blood specimens were successfully collected from 92% to 99% of children and 98% to 100% of women among those who enrolled. On the basis of experiences from these serosurveys, we identified challenges in the survey design and implementation steps that could contribute to nonsampling errors.

### Community mobilization.

Although CHWs were engaged to accompany and help field teams in all steps of field implementation, lack of cooperation from community members was observed in certain settings and populations. Limited participation was observed in affluent populations living in urban high- and middle-income settings and migrant populations living in nonpermanent or slumlike settlements. Reduced participation was most common in urban areas and often due to the lack of a relationship between the community and CHWs or local leaders. For example, in high- or middle-income apartment-style neighborhoods in urban settings, most households used private health care providers and did not have an existing relationship with the CHWs. In some urban wards, CHWs only offered services to vulnerable populations who did not have the means to access private healthcare. An important challenge in both urban and rural areas was reaching migrant populations, who were eligible for the MR campaign and serosurvey but often had a weak or no relationship with CHWs and local leaders because of the transient nature of their residence.

### Mapping of clusters.

Because the 2011 census was the most recent and complete national-level demographic record available at the time of the serosurveys, it was used for sampling purposes. However, as the census information was out of date, there were limitations to its usefulness. Outdated maps from the 2011 census contributed to two main problems. First, because administrative boundaries changed in some districts or new districts were created after 2011, input from the field investigators and local community members was needed before data collection to prepare an updated list of households in the selected village or ward. Second, there were changes to boundaries of some clusters due to rapid development and construction of new structures since 2011, which made locating the boundaries of selected clusters challenging in the field.

**Figure 2.**
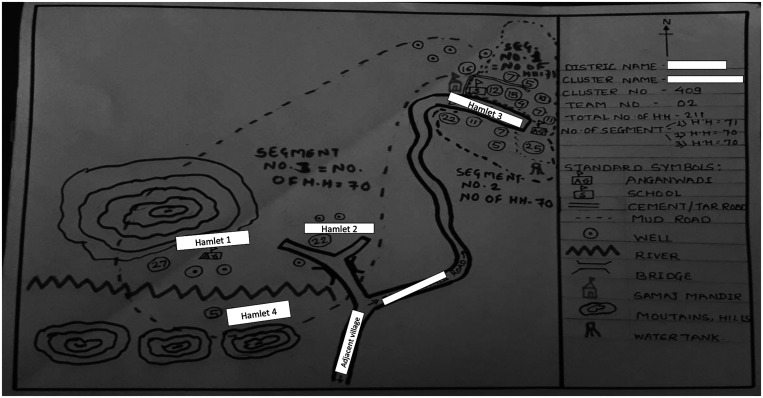
Example of a sketch map. The sketch maps are hand-drawn maps prepared by field teams to update census reference maps to reflect the current number of households in a cluster. Circle with a number indicates number of households. Thick dotted lines represent smaller segments created within sketch map in larger clusters to reduce workload while maintaining probability samples. Refer to Supplemental Appendix for more details related to segmentation.

**Figure 3.**
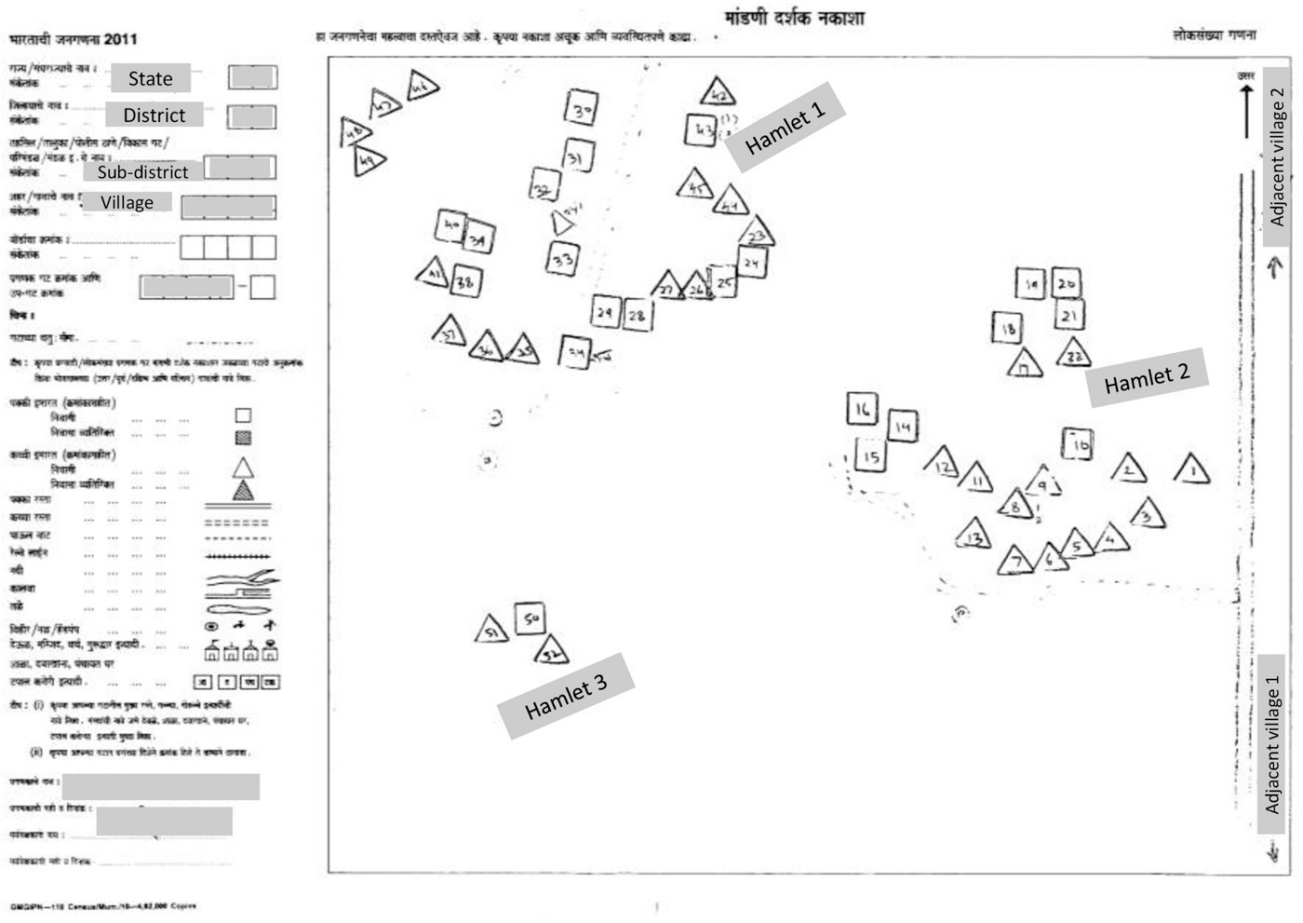
Example of a poor-quality census reference map.

Owing to the outdated census, it was important to update the sampling frame by preparing sketch maps of the clusters on the first day in the field. The sketch maps were detailed, hand-drawn maps prepared by field teams to illustrate the location and boundaries of the selected cluster, key landmarks (e.g., road names, water bodies, health centers, schools, temples) and to estimate the total number of households in the selected cluster (Figure 2). The census maps used to initially locate the boundaries of the cluster varied in quality, including some with illegible text, symbols, or landmarks; incorrect or missing information on key geographic locations; and drawings that were not to scale. Some poor-quality maps made it challenging to identify and map the selected cluster. For example, the census map of a village depicted three hamlets of houses with no landmarks or indication of distance between each hamlet (Figure 3). However, when the team prepared the sketch map of the selected cluster, they found four hamlets instead of three, which were located at long distances from each other and had a higher number of households than depicted in the census map (Figure 2). Additionally, significant geographic features were missing from the census map that would have helped the field team identify the cluster and prepare for mapping, including features like a river that separated hamlet 4 from the rest of the cluster and the presence of mountainous terrain near hamlet 1 and 4.

Nonpermanent settlements of migrant populations were not reflected in some census maps because they were new since 2011, not present at the time of the census owing to their transient nature, or excluded. Some census maps were missing or were not provided by the census office due to security issues, particularly clusters located in cantonment or military areas.

### Enumeration of households and its members.

Not all households within the selected cluster could be enumerated because they were physically inaccessible, were locked, members refused to provide information, or no competent respondent was at home. Some households were inaccessible due to challenging terrain. This was a common challenge in serosurveys conducted in remote settings where many households were difficult to access due to geographic barriers, including a body of water, agricultural land, or a mountain range. In rare situations, field teams tried to enumerate these households using information from CHWs, local leaders, other community members or by contacting families by phone. In urban communities, apartments and residential complexes often did not allow field teams to access the building or security guards were unwilling to provide information (e.g., approximate number of households) without permission from relevant authorities. Repeated visits were made to obtain permission before initiation of the fieldwork. The types of permissions needed varied by community and took time to obtain.

In other situations, households could be accessed but could not be enumerated because they were locked, they refused to provide information, or had no competent respondent (i.e., only children or elderly family members were at home). This was a common challenge in urban communities where residents were at work or school or were more likely to refuse. Across serosurveys and districts, the urban clusters on average had lower rates of successful enumeration compared with rural clusters. Other reasons, such as holidays (e.g., local festivals and summer holidays) or local events (e.g., elections and harvest), also contributed to incomplete enumeration of households.

### Enrollment of selected participants.

Participants who were selected but not enrolled in the serosurvey were either not available because their household was locked or they refused blood collection, refused for another reason, or were determined to be age ineligible at enrollment.

As described in the community mobilization section, it was challenging to gain support from affluent groups and migrant populations, particularly at enrollment. Similar to enumeration, urban clusters on average had higher nonresponse rates during enrollment compared with rural clusters. Common reasons for nonresponse at enrollment included unavailable participant (e.g., household locked) or refused blood collection. In some situations, children could not be enrolled because their age collected at enumeration was incorrect (e.g., date of birth was not or could not be verified using a reliable record) and their correct age did not meet the age eligibility criteria. Apart from individual-level reasons for nonresponse, higher nonresponse rates were observed at the cluster level due to holidays (e.g., local festivals and summer holidays) or local events (e.g., elections and harvest), similar to the situation at enumeration.

The consent procedures, a necessary step before enrollment, were logistically challenging if parents or legal guardians of younger children were at work and not physically available to provide written consent. If parents or participants were not literate, a literate witness outside the field team needed to be present, but this was often difficult in rural settings where literacy levels were low. Obtaining verbal assent from younger children (7–12 years) in addition to parent consent was challenging in some areas.

### Data collection.

Challenges faced during data collection included miscommunication of survey questions to participants, application performance (e.g., speed and Internet connectivity), and data entry errors. Miscommunication of survey questions, such as questions related to vaccination history, due to use of leading questions by field teams or the presence of CHWs (potentially leading the caregiver to exaggerate the vaccination history) sometimes influenced how the participant responded to questions. When collecting information on vaccination history for younger children, the routine vaccination card was the preferred source of data. However, in situations when the card was not available, data were collected based on parental recall, which compromised accuracy. Although MR campaign doses were recorded on a separate MR campaign card, in some sites and settings, there were reported shortages in campaign card supplies, and so the MR campaign doses were recorded on the routine vaccination card instead. Field teams recorded such doses when observed.

Using tablet-based data collection instead of paper-based questionnaires allowed for faster data entry, processing, cleaning, and analysis, with near real-time quality checks, and reduced the need to carry multiple printed questionnaires to the field. However, the complex structure of the tablet-based application (e.g., built-in calculations for determining eligibility and randomly selecting participants; potential for multiple participants and forms linked to the same household) led to performance issues. These issues resulted in inefficiencies in data collection due to lags in loading fields or forms, data duplication (e.g., same form uploaded multiple times), and data loss. Also, poor Internet connectivity in rural, tribal, and remote settings caused data upload issues that sometimes led to delays and temporary data loss. Substantial time and effort from the data managers was required to resolve issues related to data duplication or temporary data loss. Other challenges included the lack of familiarity with using tablet-based tools, which may have led to data entry errors especially when collecting dates (date of birth and date of vaccination).

### Biospecimen collection, transport and storage.

Common challenges with blood collection, transport and storage included hemolysis of the specimen and frequent power outages at the site laboratories. Hemolysis was a common problem during blood collection and transport in the community. Causes included improper collection technique or handling of the specimen at the time of collection, improper packing and transport, delayed processing, or improper storage of specimens. In these serosurveys, hemolysis occurred primarily during transport from the field due to direct contact of specimens with frozen ice packs or transport over uneven roads. There were challenges with specimen collection from younger children compared with older age groups, such as movement during collection, which likely contributed to higher rates of hemolysis in this age group. Most site laboratories were located in remote rural locations with frequent power outages, which could lead to repeated freeze–thaw cycles that can degrade antibodies. Additional challenges included maintenance of the cold chain during transport of liquid blood specimens from remote rural settings to laboratories and specimen mislabeling during specimen collection in the community and processing in the laboratory.

## DISCUSSION

Nine district-level household serosurveys were successfully implemented in India with high enrollment and specimen collection rates. On the basis of our experiences, the key challenges that can affect seroprevalence results are related to the need for reliable information to ensure a complete sampling frame, community engagement, data and specimen collection, and specimen transport and storage. To minimize the impact of these challenges, detailed recommendations by serosurvey activity are described (Table 1). These recommendations provide strategies used in serosurveys in India as well as alternative approaches to address these challenges and minimize their impact on serology results.

**Table 1:** Challenges faced and recommendations to facilitate the implementation of serosurveys

Serosurvey activity	Challenge	Recommendations
Community mobilization	Lack of cooperation from certain settings and populations*	•Establish a community mobilization plan and initiate mobilization activities prior to fieldwork. Consider the following: ^Review the list of selected clusters and compile general cluster-level characteristics such as location, urban/rural status, and presence of slum areas. ^Identify CHWs and influencers prior to serosurvey initiation. ^Account for additional time and effort required to obtain permission from affluent neighborhoods. ^Plan serosurvey timings with input from local community members to improve response rates. •Involve influencers within and outside the health system depending on setting to improve acceptance. This includes sensitizing them about the study so that they can help address questions and concerns from the community. For example, in affluent urban communities permissions from residents’ associations helped build community support for the study. whereas among migrant poor populations and minority groups, support from local WHO representatives or religious leaders was helpful. •Provide compensation to local influencers for their time to ensure sustained support. In our surveys, providing compensation to local CHWs like Accredited Social Health Activists was important because they typically receive performance and service-based compensation for their regular work. No monetary compensation was expected or provided to local or religious leaders. •Collect qualitative data on cluster level characteristics during survey conduct (e.g., urban or rural, slum or nonslum areas, and common reasons for nonresponse) to assess patterns of nonresponse across clusters. This type of data was useful for planning and interpretation of the results.
Mapping of clusters	Outdated census lists and reference maps	•Identify the most recent comprehensive list of communities for the sampling frame. If sampling is based on an outdated census, use census maps as a reference and prepare updated sketch maps of the clusters.^5^ •Engage with local leaders, health workers, and other community members to physically locate selected cluster and its boundaries.
	Variability in quality and completeness of reference maps	•Review and identify low quality and missing reference maps before field implementation. •In the case of low-quality or missing maps, use alternative spatial sampling methods or geographic units that align with cluster (depending on setting).^18^^–^^20^
Enumeration of households and its members	Inaccessible households (due to terrain or permission)	•Devote additional time and effort to include inaccessible households in enumeration such as alternate methods of contact. For example, in some situations, field teams were able to contact households that were initially inaccessible over the phone for information with support from CHWs and other community members.
	Households not enumerated because they were locked, no competent respondent at home or refusal	•Document nonresponse rates (e.g., percentage of households not enumerated) and reason for nonresponse (e.g., locked households, refusal to provide any information, or no competent respondent at home) at enumeration. •Regularly monitor nonresponse rates and adapt. For example, adjust timing based on holidays (e.g., local festivals and summer holidays) or local events (e.g., elections and harvest), with input from CHWs, or other community members supporting the field team. Be flexible with the time and day of household visits. •Report nonresponse rates at enumeration in dissemination reports and publications to provide context when interpreting seroprevalence estimates.
Enrollment of selected participants	Low participation rates due to refusal or unavailability from certain settings and populations or during certain time periods*	•Document and report nonresponse rates (e.g., percentage of selected participants not enrolled) and reason for nonresponse (e.g., locked households, refusal to provide blood, or other reason) at enrollment. •Regularly monitor nonresponse rates and reasons for nonresponse and adapt survey activities. For example, if eligible individuals refuse blood collection, increase community mobilization efforts and engage with CHWs to explain the survey procedures and address questions and concerns. •Devote additional time and effort to enroll unavailable participants, including adjusting timing of visits, scheduling follow-up visits, and returning when parents or guardians are available.
	Age ineligibility issues	•To reduce age ineligibility issues, verify date of birth or age information using reliable records (e.g., government or school record) at the enumeration and enrollment steps. If such a record is not available, verify with a reliable respondent like mother of the child.
Data Collection	Miscommunication or non-standardized administration of survey questions to participants. Low vaccination card retention and reliance on parental recall. Data entry errors (e.g., date of birth)	•Design questionnaire and data collection tools to prevent or resolve quality issues. For example, limit the questionnaire to the questions of interest and avoid extraneous questions. If feasible, electronic tablet or phone-based questionnaires with built-in validation checks for key variables and built-in skip logic can help improve data quality. •Encourage verification of key variables, such as date of birth and vaccination date, against reliable physical records such as vaccination cards, government or school identity cards or use examples based on local context when probing for recall (e.g., dates of holidays or festivals). •Other strategies include photographing vaccination cards to resolve potential data errors. •Regularly monitor data and fieldwork to identify data quality issues and provide feedback or retraining as needed. For example, near real-time data monitoring was conducted through generation of weekly cluster summary reports that highlighted cluster-level response rates, data for key variables (e.g., vaccination coverage), and potential data entry errors (e.g., date of vaccination before date of birth). These reports were circulated and discussed weekly to identify and rectify any data-quality issues. Other monitoring activities included daily oversight by site investigators, daily reporting from field teams, weekly conference calls, and frequent site monitoring visits.
	Complex infrastructure of tablet-based application and poor Internet connectivity can lead to data upload issues and temporary data loss	•Hire staff with prior experience using mobile phones or tablets. If not possible, include additional hands-on practice sessions during training and piloting. •Pilot test the functionality and performance of data collection application in the setting representative of where the survey will be conducted prior to initiation. •In case of issues with tablet, use information technology–based solutions to recover data from tablet or develop backup procedures for data collection (e.g., paper forms).
Biospecimen collection, transport, and storage	Improper blood collection in the field and improper packaging of blood specimens from field to laboratory can lead to hemolysis of specimens	•Have experienced phlebotomists collect blood from infants or younger children. Use of butterfly needles (instead of needle and syringe) may be easier when collecting blood from younger children but should only be considered for use by experienced technicians. •Assess causes of hemolysis during collection. Use recommended good practices to minimize hemolysis of blood specimens, including the following: ^Let blood specimens sit undisturbed for at least 30 minutes after collection. ^Use conditioned ice packs^†^ when storing specimens in the field to prevent the specimens from freezing leading to hemolysis. ^Centrifuge specimens in the field using a portable centrifuge before transporting, if possible. ^Carefully pack specimens for transport including using conditioned ice packs and not letting blood tubes come in direct contact with ice packs. •Monitor hemolysis in the community and laboratory after transport. Adapt procedures if needed to minimize hemolysis. •Procure supplies and equipment centrally (in one location or where the main laboratory is located) and transfer to individual sites to maintain uniform quality across multiple sites.
	Specimen mislabeling	•Reduce mislabeling issues by using of centrally generated ID numbers and preprinted labels and reconfirming ID labels in the field and laboratory.
	Improper cold chain storage and transport conditions.	•Ensure 24-hour power backup in laboratory where specimens are stored. If power backup is not available, consider transporting specimens to another laboratory nearby to ensure quality of samples. •If temperature during specimen transport is a concern, consider alternate specimen types like dried blood spots, which can be transported at room temperature.
Overall planning, coordination and logistics	Identifying implementation partners	•Identify and collaborate with experienced local implementation partners. Partners can leverage prior experiences, local knowledge, and relationships to inform all aspects of a serosurvey.
	Inadequate training of staff	•Conduct intensive in-classroom training for field teams to review background and steps of the survey objectives and methodology. Examples of helpful training strategies include the following: ^Interactive sessions involving role-play of informed consent procedures and data entry into tablet questionnaires. ^Field-based training sessions in nearby communities to practice using census maps, confirming its boundaries and mapping the area and enumeration. •Provision of supportive supervision to field teams in the early steps of the serosurvey. For example, trainers were physically present for the first cluster in each survey to supervise teams.
	Safety of teams in the field	•Consider the following steps to ensure safety of teams: ^Field teams to always enter field sites with local health workers or authorities and always inform local leaders or entities the purpose and period of their stay. If necessary, field teams can also inform local police authorities in case of anticipated issues. ^Teams must not remain in the field after sunset.
	Insufficient communication between core and field teams	•Maintain regular communication with field teams. For example, use of messaging services like WhatsApp, for connecting core team of study investigators with field teams to get regular updates from the field and address any issues in real time.
	Logistical, ethical, and budgetary challenges when returning results to participants (if applicable)	•If serology results will be returned to participants, the process, timing, ethical requirements, and budget need to be considered before the start of the survey. Additional considerations may be needed for different settings. For example, participants living in rural areas may prefer to receive results in person, whereas in urban areas via postal mail or electronically.

CHW = community health worker.

* Examples include urban high- and middle-income households/areas and migrant populations living in nonpermanent or slum settlements.

† Conditioned ice packs: ice packs that have been allowed to thaw for at least 30 minutes before use.

Outdated, poor-quality, or missing census lists contribute to incomplete sampling frames in community-based surveys. When census maps were not available or boundaries could not be identified, Anganwadi centers (AWCs), which are rural child health centers with defined geographic areas, were used as an alternative method to divide selected villages or wards. However, AWCs had variable coverage in urban areas. Using old or low-quality reference maps may lead field teams to either exclude households in selected clusters or include households from a neighboring cluster not selected for the serosurvey. If these errors in mapping are random, the reasons households are missed may not be related to their serostatus or vaccination coverage and the errors may not bias the results. However, exclusion of certain groups of households (e.g., those located in slums, in remote areas, or in gated apartment communities) can cause selection bias and impact seroprevalence estimates. If census maps are out of date or of low quality, this information could be used only as a reference and updated sketch maps should be prepared with the help of local influencers, CHWs and other community members. If feasible, consider alternative spatial sampling strategies like grid-based geographic information system sampling, which other surveys have successfully implemented in low-income settings.^15^^–^^17^

Similarly, households missed at the enumeration step could also lead to incomplete sampling frames and reduce the representativeness of findings. Because it was not always possible to document the number of households that were inaccessible (e.g., areas physically inaccessible due to terrain or buildings inaccessible for security reasons), the number of eligible members in these households, or characteristics of these individuals, we were unable to monitor or report the size or direction of the potential systematic error. However, for households that were accessible but could not be enumerated, it was possible to document the number of households missed and the reason why they could not be enumerated. High nonresponse rate at either enumeration or enrollment steps can increase random error, which affects the precision of seroprevalence estimates or causes selection bias if nonresponse is disproportionately high in certain communities. Hence, nonresponse rate and reason for nonresponse (e.g., refusal, locked household, availability of the participant) should be documented at both the enumeration and enrollment steps. This will help estimate the magnitude of nonresponse, and regular monitoring and reporting of nonresponse can help inform the field teams on strategies to improve participation. This information was particularly useful in the initial clusters of the surveys; however, nonresponse rates typically decreased as teams gained more experience, with the exception of some challenging clusters.

Lack of community support can result in incomplete sampling frames (e.g., refusals during enumeration) and reduce response rates of selected participants. To improve community engagement, teams should establish and initiate a community mobilization plan, including involvement of local influencers. This can include monitoring of cluster-level characteristics to assess patterns of nonparticipation across clusters.

Data collection issues can be minimized with simple solutions like limiting the questionnaire to the primary questions of interest and not collecting extraneous information. If feasible, electronic questionnaires with built-in validation checks for key variables and skip logic can minimize common data collection errors. Teams should verify key variables such as birthdate and vaccination date against reliable records such as vaccination cards and government or school identity cards. If case records are not available, data collectors can use examples from the local context, including dates of local holidays or events, when probing by recall. A poorly completed vaccination card with illegible data may result in under- or overestimating vaccination coverage depending on how field teams interpret the records. If the vaccination card was missing and vaccination history was collected verbally, the parents might overreport due to social desirability bias or influence of the CHW or underreport due to lack of information about the child’s vaccination status.^18^^–^^20^ Inaccurate coverage data in either direction would influence how the seroprevalence results were interpreted. Other surveys have also reported photographing vaccination cards to help resolve data errors without needing to return to the field.^10^^,^^21^

The impact of missing data is dependent on the type and magnitude of data missing. If a key variable such as age is missing, then the record may need to be dropped from analyses or statistical methods used to impute the value. Data upload errors resulting in data loss for an individual or an entire survey cluster, particularly those related to survey setting (e.g., remote and rural), will undermine the representativeness of the sample and could bias estimates. Similar to data loss, data entry errors will have a larger impact if key variables are affected. For example, if the date of birth of an enrolled child is incorrectly recorded, then that will have implication on age-specific seroprevalence estimates. Hence, near real-time monitoring of data (e.g., field monitoring and weekly data reports) should be implemented with efficient feedback systems to the field sites (e.g., frequent calls and messaging services such as WhatsApp for daily communications with field teams) to identify data issues during the surveys. If using an electronic tablet or phone-based data collection application, there may be additional considerations when hiring staff and having in place information technology–based solutions or backup procedures for data collection.

Although antibodies are relatively stable to changes in temperature, proper handling of samples and maintenance of the cold chain is important in generating accurate results. The selected enzyme immunoassay was reportedly not affected by hemolysis according to the manufacturer; however, it is important to minimize antibody degradation, which can systematically underestimate seroprevalence. Hemolysis during collection can be minimized by hiring experienced phlebotomists and using butterfly needles when drawing blood from young children. Other good practices include using conditioned ice packs (ice packs that have been allowed to thaw for a few minutes before use) to prevent freezing and subsequent hemolysis of blood specimens, using a portable centrifuge in the field before transport, and careful packaging of samples in ice-pack-lined cold boxes. Improper cold chain storage conditions can be avoided by ensuring 24-hour power backup at the storage facility. If this is not possible, consider transporting specimens to another facility where it is feasible. Teams can also consider alternative specimens including dried blood spots collected by finger-prick on filter paper, which will help minimize hemolysis and cold chain challenges related to blood collection, transport, and storage.^22^^–^^25^ Mislabeling of specimens can lead to mismatches between participant information and laboratory data. If there is mislabeling on a large-scale, this may also affect interpretation of age-specific seroprevalence estimates. Specimen mislabeling errors can be reduced by using centrally generated identification numbers and preprinted specimen labels.

Other common challenges for serosurveys include identifying implementation partners, inadequate training of staff, safety of teams in the field, and insufficient communication between the core and field team. If serology results will be returned to participants, additional logistical, ethical, and budgetary challenges should be considered before starting the survey. Although these challenges may not have a clear impact on systematic and random errors and study outcomes, taking them into account when designing and implementing the survey may help improve participation and overall quality.

These recommendations may add time and cost to the serosurveys but may be further adapted depending on what is feasible in other settings. There are alternative approaches to population-based serosurveys that may be less logistically challenging, such as nesting a serosurvey within a larger survey or using residual specimens from other surveys, research studies, surveillance systems, or health facilities.^2^^,^^26^^–^^28^ Although these alternative approaches may be easier to conduct, there may be other challenges to be considered, such as representativeness of specimens.

The lessons learned from these serosurveys in India can help generate high-quality estimates of seroprevalence, which are increasingly needed for vaccination and disease control programs as more countries move toward elimination goals and targeted vaccination strategies.

## Supplemental Material


Supplemental materials
