# Supplementary material for: Implementing Serosurveys in India: Experiences, Lessons Learned, and Recommendations
Source: Am J Trop Med Hyg. 2021 Oct 4;105(6):1608–17. doi: 10.4269/ajtmh.21-0401 (PMC8641364; doi:10.4269/ajtmh.21-0401)
Supplement: Supplementary file 1 [file tpmd210401.SD1.pdf]

Supplementary methods

Study design and sampling strategy

Nine cross-sectional serosurveys were conducted in five districts in India. This included four districts where pre and post measles and rubella (MR) campaign serosurveys and 1 district where only the post MR campaign serosurvey was completed. Four districts were selected based on geographic diversity, presence of an established Model Rural Health Research Unit (MRHRU), and the ability to conduct serosurveys before and after the MR vaccination campaign. MRHRUs are government research facilities located in rural areas of India set up in 2013 by the Government of India to promote health research activities among rural populations. One additional district was selected to reflect a setting with use of rubella vaccine in the private sector. The surveys were conducted among three age groups (9 months to less than 5 years, 5 to less than 15 years, and women 15 to less than 50 years [post MR campaign only]).

Using guidance from the WHO Vaccination Coverage Cluster Survey Reference manual and Demographic and Health Survey manuals, a three-stage cluster design was adopted in which first villages and wards were selected, then one cluster or Census enumeration Block (CEB) was randomly selected from each village and ward, and third, all age-eligible individuals in the selected cluster were enumerated and then randomly selected for the study. In the first stage, in each district 30 villages in rural areas or wards in urban areas were selected based on the 2011 nationwide census using the probability proportional to size. For the post campaign serosurveys, new clusters were selected to minimize the chances of enrolling the same participants and thus reduce potential bias introduced after the initial survey interaction. In the second stage, from each village or ward, one cluster was randomly selected and then all individuals in the cluster were enumerated. The generic term “cluster” was used for a CEB which as per the India census is a well-defined area in a village or ward with 120-150 households (approximately 600-750

population) (Census of India, 2011) allotted to an enumerator at the time of the decennial census. In the third stage, thirteen eligible individuals per age group were selected by simple random sampling.

Field implementation

The survey team identified the selected cluster and verified its boundaries with the help of community health workers (CHWs) using census maps procured from the regional census offices. Wherever census maps were either not available or did not have any clear landmarks, Anganwadi centers were used instead of clusters. One Anganwadi center caters to a population of 300-800 people to provide basic nutrition, health, and early education services. We listed the Anganwadi centers in the village/ward and randomly selected one Anganwadi center as an alternative to the CEB.

After identifying the selected cluster and its boundaries, the team conducted a rapid mapping exercise to count the number of households and assessed if segmentation of the cluster was needed for logistical reasons. A threshold of 70 households was set to ensure at least 13 individuals per age group were present in the final study cluster. This threshold was based on birth rates, infant mortality ratio (estimated from the Sample Registration System), and household size (estimated from the Census of India, 2011). If there were 70-140 households in the cluster no segmentation was done. If there were more than 140 households, the cluster was segmented and one segment was randomly selected by an independent statistician at ICMR-National Institute of Epidemiology, Chennai.

The survey team then enumerated all households in the final study cluster, such that each household was numbered and demographic characteristics of people residing in the households, including name, age, sex and availability in the next 3 to 4 days, were collected using an Android tablet-based application. All available age-eligible children and women

enumerated from the cluster constituted the final sampling frame. The enumeration data were uploaded to the server of at Indian Council of Medical Research-National Institute of Epidemiology (ICMR-NIE), Chennai and 13 individuals per age group were randomly selected centrally.

The survey team then conducted enrollment by visiting all selected individuals to collect data on vaccination status and household-level socio-demographic details and a blood specimen, after obtaining consent or assent. Up to three household visits were made to enroll selected individuals.

The Institutional Ethics Committees of all the participating institutes and Johns Hopkins Bloomberg School of Public Health approved the study protocol. Ethical approval was obtained in the form of written informed consent (people aged 18 years and older), parental consent (for children less than 18 years) and assent (children between 7 to less than 18 years) before data or specimens were collected.

Biospecimen collection, transport, storage, testing and analysis

Up to 2 mL of venous blood was collected in a serum separator tube (Becton Dickinson 367983). Samples were left at ambient temperature for 30 minutes after collection, centrifuged at 3000 revolutions per minute for 10 minutes using a portable centrifuge, and stored at 4-8° C in cold boxes until transported back to the site laboratory at the end of the day. In the laboratory, specimens were re-centrifuged, sera were aliquoted and stored at -20° C. At the end of the survey, sera were transported to the ICMR- National Institute of Virology, Pune under cold-chain for serologic testing.

All sera were tested using commercial anti-measles IgG and anti-rubella IgG enzyme immunoassay kits. Equivocal samples were retested in duplicate using the same assay and the qualitative result most commonly observed out of the three results was selected as final.
